# Preprocedural Hypertension Is Not a Risk Factor for Postoperative Bleeding following Image-Guided Core Needle Breast Biopsy

**DOI:** 10.1155/2021/9634938

**Published:** 2021-09-06

**Authors:** Ninad Salastekar, Alexis Saunders, Kushal Patel, Katherine Willer

**Affiliations:** Department of Radiology, SUNY Upstate Medical University, 750 East Adam Street, Syracuse, NY 13210, USA

## Abstract

**Objective:**

To evaluate the association between preprocedural hypertension and the risk of prolonged bleeding following image-guided core needle breast biopsy in nonpregnant/nonlactating women.

**Methods:**

A single institution-based, retrospective cohort study of 400 women who underwent image-guided core needle breast biopsy was conducted. Males and pregnant and lactating women were excluded. Preprocedural systolic or diastolic blood pressure greater than 140 or 90 mm of Hg, respectively, was defined as hypertension. Prolonged bleeding was defined >15 minutes of local, manual pressure required to achieve hemostasis following the biopsy. Severe bleeding complications defined as clinical significant hematoma formation, prolonged bleeding requiring an ER visit, hospitalization, or surgical intervention were also recorded.

**Results:**

The difference in the mean time for which manual pressure was held after biopsy for patients with and without preprocedural hypertension was not statistically significant (13 ± 7 vs. 13 ± 8 minutes, respectively, *P* = 0.856). There was no difference in the number of patients requiring manual postoperative pressure >15 minutes between those with preprocedural hypertension and the normotensive patients (13% vs. 12%, respectively, *P* = 0.765). Bivariate analysis demonstrated statistically significant association between prolonged bleeding and current antithrombotic or antiplatelet medication use (*P* = 0.010), the use of stereotactic guidance (*P* = 0.019), and a tethered vacuum-assisted device (*P* = 0.045). The use of a tethered vacuum-assisted biopsy device was the only variable associated with prolonged bleeding in the multivariate model (*P* = 0.044).

**Conclusion:**

Preprocedural hypertension is not a risk factor for prolonged bleeding following image-guided core needle breast biopsies in nonpregnant/nonlactating women.

## 1. Introduction

The lifetime risk of developing breast cancer for an average woman in the US is 12.9%, and approximately 128 new cases of breast cancer are diagnosed per 100,000 women per year [1]. Image-guided core needle breast biopsy (CNB), an accurate and cost-effective alternative to open surgical biopsy, remains the investigation of choice for further evaluation of a majority of suspicious findings discovered on breast imaging [2–4]. Core needle breast biopsies are associated with a low complication rate and are generally well tolerated by patients [2, 3]. Overall, the rate of severe complications from CNB procedure is <1% [5]. While a systematic review by Bruening et al. reported bruising, vasovagal reaction (3.0%), and pain (3.7%) as the most common minor complications, severe bleeding complications (prolonged/severe bleeding requiring treatment (0.7%) and postoperative hematoma formation (0.1%)), infections (0.15%), and severe pain (1.7%) were reported as the most common major complications following CNB [3].

While the incidence of severe bleeding complications (SBC) following CNB is low, the risk factors associated with these complications remain poorly understood. Vacuum-assisted biopsy device is associated with increased risk of SBC following CBC, while antithrombotic medication use is not [3, 6, 7]. There is insufficient evidence to demonstrate the association of patient comorbidities with the risk of SBC [3]. Particularly, systemic hypertension is a known risk factor for SBC following kidney biopsy [8, 9]. However, to the best of our knowledge, the association of systemic hypertension with severe bleeding complications following core needle breast biopsy has not been studied. In this study, we evaluate the association between systemic hypertension and the risk of SBC following CBC in women with suspicious findings on breast imaging.

## 2. Materials and Methods

The institutional review board approved this single institution-based, retrospective cohort study conducted at a university hospital. 400 consecutive women who underwent ultrasound (US) or stereotactic-guided core needle breast biopsy from January 2016 to June 2020 were included in this study. Exclusion criteria included concurrent pregnancy or lactation and male gender. Data were acquired through chart review.

The past medical history of the subjects was reviewed to note any hematological disorders that could affect hemostasis as well as history of systemic hypertension. Medications including antithrombotic and antiplatelet agents were noted. In accordance with currently accepted clinical guidelines, antithrombotic/antiplatelet therapy was not held prior to procedures, neither was the coagulation profile (INR/PT) acquired immediately prior to the procedure. The type of biopsy (US/Stereotactic), biopsy device (vacuum-assisted or not), number of biopsy sites, and number of biopsies per site were recorded. Preprocedural blood pressure was noted for each subject. Preprocedural systolic or diastolic blood pressure greater than 140 or 90 mm of Hg, respectively, was defined as hypertension.

The main outcome of interest was prolonged bleeding as measured by the time for which manual pressure was applied to the biopsy site to achieve hemostasis. Prolonged bleeding was defined as greater than 15 minutes of pressure required to achieve hemostasis. Severe bleeding complications defined as clinically significant hematoma formation, prolonged bleeding requiring an ER visit, hospitalization, or surgical intervention were also recorded.

## 3. Statistical Analysis

Analysis was conducted using R 4.0.2 and RStudio [10, 11]. Continuous variables (age and time to hemostasis) were described as mean ± standard deviation. Categorical variables were described as frequency (percentage). Bivariate analysis was conducted using the independent *T*-test/Mann–Whitney test for continuous variables and the Chi Square test/Fisher's exact test for categorical variables. Variables with *P* value <0.10 were included in a multivariable logistic regression model. A *P* value of 0.05 was considered as the threshold for statistical significance.

## 4. Results

The demographic and relevant clinical characteristics of the patients are described in Table 1. The average age of patients in this study was 59 ± 13 years. Approximately 2% patients had a history of hematological disorders that could potentially affect hemostasis (for example, antiphospholipid syndrome and protein C/S deficiency), 39% had a history of hypertension, and 11% had a history of current or past smoking. About 5% patients in the study were taking antithrombotic/antiplatelet medications (warfarin, direct thrombin inhibitors, and clopidogrel). 77% of all biopsies were ultrasound-guided, and the remaining were stereotactic biopsies. All stereotactic biopsies used a tethered vacuum-assisted biopsy device. Needles with ≥12 gauge were used in 68% of the biopsies. 31% of patients in this study had preprocedural hypertension, defined as a systolic or diastolic blood pressure greater than 140 or 90 mm of Hg, respectively. The distribution of patient characteristics grouped by presence of preprocedural hypertension is also described in Table 1.

A total of 137 subjects were missing information regarding bleeding outcomes and 68 were missing information regarding preprocedural blood pressure.

The distribution of the systolic and diastolic blood pressures in patients stratified by preprocedural hypertension and prolonged bleeding is shown in Table 2.

The difference in the average time for which manual pressure was held after biopsy for patients with and without preprocedural hypertension was not statistically significant (13 ± 7 vs. 13 ± 8 minutes, respectively, *P* = 0.856, Table 3). There was no difference in the number of patients requiring manual postoperative pressure >15 minutes between those with preprocedural hypertension and the normotensive patients (13% vs. 12%, respectively, *P* = 0.765, Table 1).

Bivariate analysis demonstrated statistically significant association between prolonged bleeding and current antithrombotic or antiplatelet medication use (*P* = 0.010), the use of stereotactic guidance (*P* = 0.019), and a tethered vacuum-assisted device (*P* = 0.045) (Table 3). Type of biopsy, use of tethered vacuum-assisted biopsy device, and anticoagulant use were further evaluated in a multivariate logistic regression model for association with prolonged bleeding (>15 minutes). The use of a tethered vacuum-assisted biopsy device was the only variable associated with prolonged bleeding in the multivariate model (*P* = 0.044).

A single patient required a visit to the ER for prolonged bleeding but was discharged without intervention. No clinically significant hematomas were reported.

## 5. Discussion

Prolonged bleeding in patients with preprocedural hypertension undergoing biopsies of the kidney has been reported [8, 9]. To the best of our knowledge, the association of systemic hypertension with severe bleeding complications following image-guided core needle breast biopsy has not been adequately evaluated.

In the current study, we found no association between preprocedural hypertension and prolonged bleeding following image-guided core needle breast biopsy (CNB). A history of systemic hypertension was also not associated with prolonged bleeding following CNB. Factors associated with prolonged bleeding, defined as >15 minutes of manual and postoperative pressure required to achieve hemostasis, were the current use of antithrombotic/antiplatelet medications, stereotactic-guided biopsies, and the use of a tethered vacuum-assisted device. A single patient with preprocedural hypertension (207/70 mm Hg), not anticoagulated, undergoing ultrasound-guided breast biopsy was transferred to the ER for prolonged bleeding, but was eventually discharged without the need for surgical intervention or hospitalization. There were no other reported severe bleeding complications in the study cohort (including clinically significant hematoma, ER visit/hospitalization, or surgical intervention).

The findings of prolonged postoperative bleeding in patients undergoing stereotactic biopsies/using tethered vacuum-assisted devices are similar to those reported previously [3, 5]. We report increased incidence of prolonged bleeding, defined as >15 minutes of pressure required for hemostasis, in patients taking antithrombotic/antiplatelet medications. However, none of the patients reported clinically significant postoperative hematomas or bleeding requiring intervention/hospitalization. Previous studies have used different criteria for defining bleeding complications but have also not reported clinically significant hematomas or severe bleeding complications in patients using antithrombotic medications [7]. Multivariate analysis demonstrated a significant association between prolonged bleeding and the use of a tethered vacuum-assisted device, a finding consistent with previous reports [5].

This study has several limitations. Findings from this single institution study have not been externally validated. MRI-guided biopsies were not included. Factors that could affect bleeding such as platelet count and INR were not recorded for a majority of patients on the day of the biopsy due to absence of such a requirement in the clinical guidelines for image-guided core needle breast biopsy. Alcohol intake on the day of the procedure was not recorded may confound the bleeding time. The study could not evaluate the effect of operator experience or skill since all the biopsies were performed by attending radiologists without the involvement of residents or fellows. The type of lesion/pathology was not accounted for. However, there is no evidence in the literature that postoperative bleeding is reliably affected by the type of pathology. Data regarding the number of biopsy sites and/or the number of biopsies per site were not consistently recorded in the original reports and thus could not be included in the analysis. However, there is no evidence to suggest a differential distribution of the number of biopsies in patients with/without preprocedural hypertension, and as such, this is unlikely to affect the results. This study did not consider the effect of patient positioning which may affect postbiopsy bleeding rates. We may have missed any severe bleeding complications that were not reported to our hospital and in cases where the patients sought care elsewhere.

## 6. Conclusion

A history of systemic hypertension and/or preprocedural hypertension is not a risk factor for prolonged bleeding following image-guided core needle breast biopsies in nonpregnant/nonlactating women. Despite the described limitations, the findings of this study should help guide clinical management of hypertensive patients on the day of the breast biopsy, specifically avoiding unnecessary delay or postponement of the biopsy due to perceived risk of prolonged bleeding in patients with preprocedural hypertension. The findings also inform the relatively unclear literature on the patient-related risk factors for prolonged bleeding following breast biopsies.

## Figures and Tables

**Table 1:** Demographic and clinical characteristic of subjects by preprocedural hypertension.

	All patients (*n* = 332)	Preprocedural hypertension (*n* = 104)	Normotensive (*n* = 228)	*P* value ^
Age^*∗*^ (in years)	59 (13)	63 (13)	57 (12)	<0.001^
History of smoking (current or past)^*∗∗*^	37 (11%)	4 (4%)	33 (15%)	0.004 ^
History of hypertension^*∗∗*^	131 (39%)	55 (53%)	76 (33%)	<0.001^
History of hematological disorders affecting hemostasis^*∗∗*^	6 (2%)	1 (1%)	5 (2%)	0.435
Antithrombotic/antiplatelet medications^*∗∗*^	17 (5%)	8 (8%)	9 (4%)	0.151
Type of biopsy^*∗∗*^				
Ultrasound-guided	251 (77%)	79 (77%)	172 (77%)	0.895
Stereotactic	81 (24%)	24 (23%)	57 (25%)	0.690
Tethered vacuum-assisted device^*∗∗*^	76 (23%)	25 (24%)	51 (23%)	0.768
Needle gauge ≥12^*∗∗*^	180 (68%)	59 (71%)	121 (67%)	0.531
Bleeding after biopsy (in minutes)^*∗*^	13 (8)	13 (7)	13 (8)	0.856
Prolonged postbiopsy bleeding (>15 minutes)^*∗∗*^	32 (12%)	11 (13%)	21 (12%)	0.765

^*∗*^Mean (standard deviation). ^*∗∗*^Frequency (percentage). ^^^Statistically significant *P* value (<0.05).

**Table 2:** The distribution of the systolic and diastolic blood pressures stratified by preprocedural hypertension.

	Preprocedural hypertension (*n* = 104)	Normotensive (*n* = 228)	*P* value	Prolonged bleeding (*n* = 32)	Normal hemostasis (*n* = 231)	*P* value
Systolic blood pressure (mm of Hg)^*∗*^	152 (14)	121 (11)	<0.001^	131 (17)	131 (18)	0.944
Diastolic blood pressure (mm of Hg)^*∗*^	87 (8)	77 (7)	<0.001^	81 (9)	80 (9)	0.461

*∗*Mean (standard deviation). ^^^Statistically significant *P* value (<0.05).

**Table 3:** Bivariate analysis of factors affecting postbiopsy bleeding time.

	Prolonged bleeding (*n* = 32)	Normal hemostasis (*n* = 231)	*P* value ^
Age^*∗*^ (in years)	59 (11)	58 (14)	0.895
History of smoking (current or past)^*∗∗*^	2 (6%)	26 (12%)	0.356
History of hypertension^*∗∗*^	11 (34%)	87 (38%)	0.719
History of hematological disorders affecting hemostasis^*∗∗*^	1 (3%)	5 (2%)	0.733
Antithrombotic/antiplatelet medications^*∗∗*^	5 (16%)	10 (4%)	0.010 ^
Type of biopsy^*∗∗*^			
Ultrasound-guided	22 (71%)	191 (84%)	0.070 ^
Stereotactic	11 (34%)	39 (17%)	0.019 ^
Tethered vacuum-assisted device^*∗∗*^	10 (31%)	38 (17%)	0.045 ^
Needle gauge ≥12^*∗∗*^	16 (59%)	139 (77%)	0.051
Preprocedural hypertension	11 (34%)	73 (32%)	0.765

^*∗*^Mean (standard deviation). ^*∗∗*^Frequency (percentage). ^^^Statistically significant *P*value (<0.05).

## Data Availability

The clinical data used to support the findings of this study are restricted by the institutional review board in order to protect patient privacy. Data are available from Ninad Salastekar (ninad.salastekar@gmail.com) for researchers who meet the criteria for access to confidential data.
